# Arthroscopic Capsular Release for Post-traumatic Elbow Stiffness

**DOI:** 10.7759/cureus.47838

**Published:** 2023-10-27

**Authors:** Kassem Ghayyad, Zahra Ahmadi, Hadi Rajabi, Mohammad M Alimohammadi, Amir R Kachooei

**Affiliations:** 1 Orthopedic Surgery, Rothman Orthopaedics Florida at AdventHealth, Orlando, USA; 2 Orthopedic Research Center, Mashhad University of Medical Sciences, Mashhad, IRN

**Keywords:** minimal clinically important difference, patient-reported outcomes, arthroscopic release, elbow stiffness, post-traumatic

## Abstract

Background: Post-traumatic elbow stiffness (PTES) may substantially affect the patient's functional range of motion and quality of life. Open elbow release has been extensively studied, but arthroscopic techniques are limited, particularly in differentiating between post-traumatic and non-traumatic stiffness. The purpose of this study is to assess the clinical outcomes after arthroscopic release of PTES regarding the range of motion (ROM), pain, functional assessment, and complications.

Methods: A prospective cohort was conducted on adult patients who underwent arthroscopic arthrolysis for PTES, with 32 patients included in the final analysis. The ROM was measured using the orthopedic goniometer. Grip strength was measured using the Camry digital hand dynamometer (Camry, CA, USA) and compared to their contralateral side. The functional status of the patients was evaluated using the American Shoulder and Elbow Surgeons Score (ASES)andthe Mayo Elbow Performance Index (MEPI). All measurements were done before surgery and at the last follow-up visit. Pre-operative and post-operative changes in MEPI, ASES, and visual analog (VAS) scores were compared with the paired t-test.

Results: After surgery, the ROM significantly improved from 74 ± 11 to 110 ± 15 degrees (p<0.001). Additionally, the ASES score and MEPI index both significantly improved from 69 ± 3.4 to 79 ± 6.3 and from 64 ± 5.7 to 82 ± 8, respectively (p<0.001). VAS scores also significantly improved from 1.1 ± 0.87 to 0.31 ± 0.53 at rest (p<0.001). The complication rate was 12%, including three transient ulnar nerve paresthesia and one superficial infection. Post-traumatic elbow release was more offered in distal humerus fractures (53%), followed by proximal ulna fracture/dislocations (25%).

Conclusion: We believe that arthroscopic arthrolysis is a safe and reliable treatment of PTES, which improves joint visibility and reduces pain. Patients can be counseled regarding the risk of a secondary surgery following distal humerus or proximal ulna fractures, including the expected recovery and complication rate.

## Introduction

The normal adult elbow range of motion (ROM) varies considerably from -21° to 12° in extension and 122° to 165° in flexion [1]. Elbow stiffness is historically a flexion-extension arc of <100°, <50° of pronation, and <50° of supination, or flexion contracture of >30° [2]. The incidence of post-traumatic elbow stiffness is reported to be up to 5% [3], where high-energy trauma and longer immobilization are correlated with more severe contracture [4,5].

An effective treatment strategy for post-traumatic elbow stiffness (PTES) entails regaining the functional range and stability of the elbow [6]. Non-operative treatment options include heating methods, myofascial mobilization, corrective splinting, and passive/active ROM [4]. Despite advances in non-operative measures, approximately 12% of traumatic elbow injuries result in PTES requiring surgical intervention [7]. Also, contemporary activities such as bringing a cell phone to the ear require more flexion to 148°, and keyboard typing requires more pronation to 65°, which expands the window of surgical treatment [8]. Patients who fail to improve after six to 12 months of non-operative measures are candidates for open or arthroscopic elbow releases [9-12]. Numerous studies on open elbow release reported good outcomes after traumatic and degenerative elbow contracture [13]. However, studies on arthroscopic elbow release, especially those separating post-traumatic from non-traumatic are limited [9,12,14-20]. Open versus arthroscopic elbow release results have shown a comparable gain in motion (51° vs. 40°) with a lower risk of complications with arthroscopic surgery (23% vs. 5%) [21]. The average motion improvement after post-traumatic arthroscopic elbow release ranges from 18° [16] to 66° [9], which is directly correlated with the pre-operative severity of motion limitation. It should be considered that the arthroscopic elbow release is demanding and is best indicated for contractures without neuropathy or extensive heterotopic ossification. A limiting factor is the reduced intra-articular volume that restricts instrument motion, obscures visualization, and puts nerves and vessels at risk of iatrogenic injury. Arthroscopic elbow releases are best performed by surgeons with experience who are aware of special techniques, including the use of retractors to add visualization and permit to address a more complex contracture safely [18,22-24].

This study aims to assess the clinical outcomes following arthroscopic release of PTES regarding ROM, pain, functional assessment, and complications. This article was previously presented as an abstract at the Mid-Atlantic Shoulder and Elbow Society (MASES) meeting held on September 9, 2023.

## Materials and methods

Study design

The study was approved by the Ethics Committee of Mashhad University of Medical Sciences (IR. MUMS. MEDICAL.REC.1397.700). The prospective cohort included adult patients with PTES treated with arthroscopic release between March 2016 and 2020. Patients with non-traumatic elbow stiffness such as osteoarthritis and severe bony deformity such as malunion requiring osteotomy and nonunion requiring open surgery were excluded. During the study period, 33 patients were operated on for PTES. We assessed the patients pre-operatively with plain radiographs and computed tomography scans if indicated. CT scans were taken to assess the location of the osteophytes for pre-operative planning and ordered per the surgeon’s discretion. Of 33, 32 patients (21 men, 11 women) were available at the final follow-up.

Measurements were done before surgery and at the last follow-up visit. The ROM was measured using the orthopedic goniometer. Grip strength was measured using the Camry digital hand dynamometer (Camry, CA, USA) and compared to their contralateral side. The functional status of the patients was evaluated using the American Shoulder and Elbow Surgeons Score (ASES) and the Mayo Elbow Performance Index (MEPI), which assesses pain, ROM, stability, and function. Pain was assessed on a Visual Analog Scale (VAS) pre-operatively and post-operatively. The minimal clinically important difference (MCID) was calculated using the distribution-based method of half the standard deviation of the post-operative changes for ROM, ASES, MEPI, and VAS.

Surgical technique

**Figure 1:**
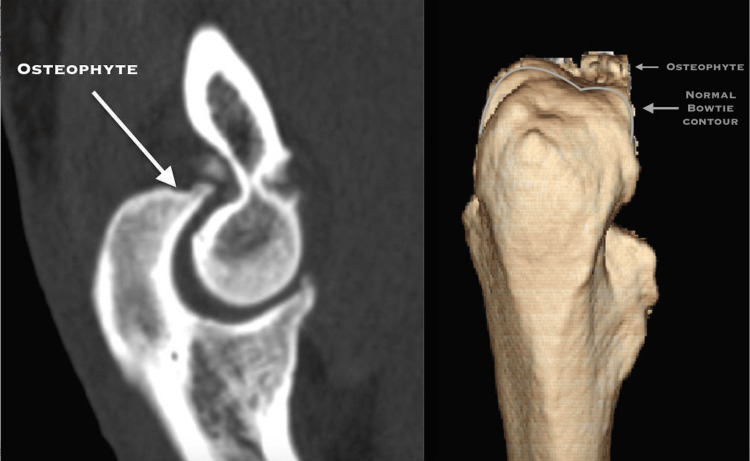
A preoperative CT scan of the elbow locating the impinging osteophytes Original work of authors

**Figure 2:**
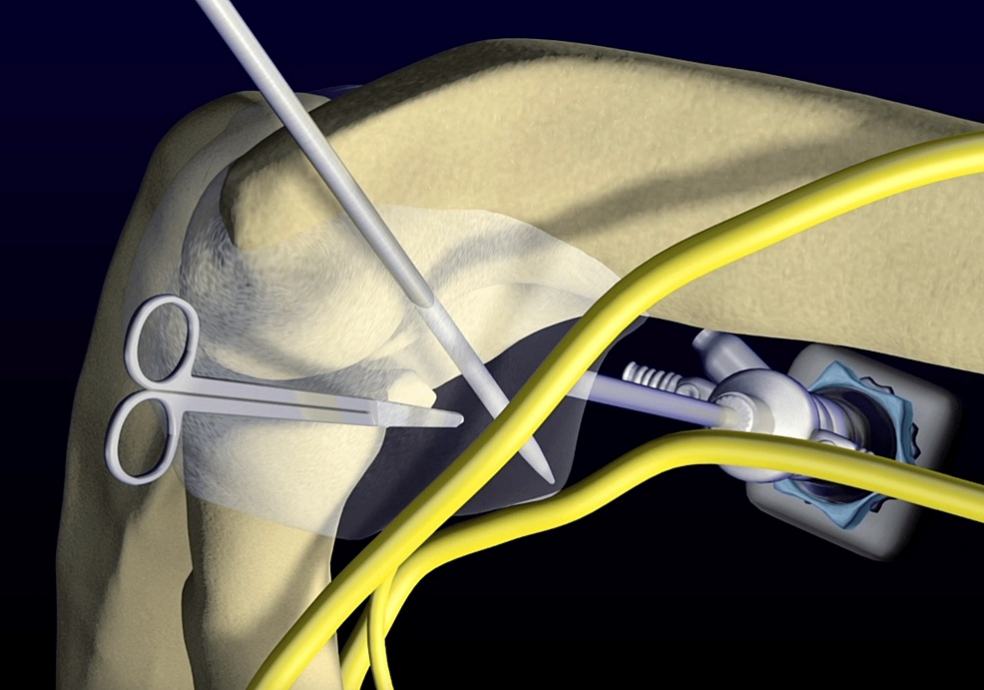
The use of a retractor for better visualization Original work of authors

All surgeries were performed by one fellowship-trained elbow surgeon (AK) under general anesthesia in a lateral decubitus position with an arm tourniquet. Our technical recommendations based on our experience with post-traumatic elbow arthrolysis are summarized in Table 1 and Figures 1-2.

**Table 1:** Recommendations for post-traumatic elbow arthrolysis

General
Pre-operative ROM will increase or remain unchanged, with no loss of motion in any direction
A Pre-operative CT scan is recommended to locate the impinging osteophytes (Figure 1)
To find and address residual obstacles, dynamic arthroscopy is advised
After completion of arthroscopic release, elbow manipulation is recommended to break residual adhesions
When using a pump, it is better to set it at the lowest rate to delay extravasation which obscures visualization
Posterior Compartment
The olecranon fossa is the safest spot to start the arthroscopy because it is filled with fibrous adhesions that obscure visualization
Shaving inside the olecranon fossa can sometimes be started blindly to free up the space and allow the camera to find the working tool
The floor of the ulnar nerve is an important part of the capsule that should be debrided until the ulnotrochlear joint line is visualized
The ulnar nerve is the closest to the joint capsule, and the shaver should always face the bone to shave the capsular attachment rather than the capsule
When addressing radial head rotation, the posterolateral portal is the viewing portal, and the direct posterior portal is the working portal
Driving through the bare area into the ulno-trochlear joint can be indicative of posterolateral instability
Anterior Compartment
If the anterior humerus bone requires shaving, the anteromedial portal is better to be placed from outside-in and slightly anterior to the humerus rather than parallel to it
More proximal anteromedial and anterolateral portals are more helpful for post-traumatic elbow arthrolysis
An anterior capsule incision along the joint line is more effective for restoration of motion than sweeping a pin off the proximal attachment to the humerus
To avoid multiple incisions, switch portals and incise the anterior capsule last. Keep the capsular tent intact for safe working and better visualization (Figure 2)

After marking the landmarks, the joint was insufflated with 10-20mL of saline via the posterocentral portal into the olecranon fossa. Posterior compartment arthrolysis was performed first using the posterocentral and direct posterior portals as the working portals and the posterolateral accessory portal as the viewing portal. Olecranon fossa, posteromedial, and posterolateral of the elbow were cleaned using a shaver and coblation. With caution, the cubital tunnel was cleaned, and the posteromedial capsule and posterior band of the medial collateral ligament (pMCL) were released under the ulnar nerve using a shaver facing the bone while the nerve was protected behind the shaver. We cleaned this area until the ulnotrochlear joint line visualization was completed, and the shaver could be driven down the tunnel while the nerve was located behind the shaver.

Through the posterolateral accessory portal, the bare area, the greater and lesser sigmoid notches, were cleaned and visualized using a shaver through the direct posterior portal. The posterior radiocapitellar joint was cleaned, and the proximal edge of the annular ligament was shaved to see the free rotation of the radial head. Osteophytes and extra bones were removed using the burr or mini osteotomes. Dynamic arthroscopy was performed to check for residual impingement.

After adequate posterior compartment debridement, we approached the anterior compartment via anteromedial and anterolateral portals. With caution, the anterolateral portal was established for visualization, and the anteromedial portal was created using the inside-out method with the switching stick through the anterolateral portal. Debridement of adhesions and osteophytes was done using a shaver and burr. The anterior release was followed by anterior capsulotomy, and capsulectomy dynamic arthroscopy was performed to check for residual impingement. After adequate debridement of the posterior and anterior compartments, the ROM was examined, and manipulation was performed to regain motion.

Post-operative protocol

All patients received indomethacin 75 mg daily for six weeks and the sutures were removed at two weeks post-operatively. Physical therapy, beginning one day after surgery focused on flexion for the first two weeks then with full range of motion for 2 to 3 months. Sixteen patients who had a range of motion less than the functional range of 30-130 degrees were given an intra-articular corticosteroid injection with continued physical therapy at three months follow-up. Injection included 3 cc’s of 2% lidocaine, and 2 cc’s of triamcinolone.

Statistical analysis

Pre-operative and post-operative changes in MEPI, ASES, and VAS scores were tested by the paired t-test. Statistical differences were considered significant for p values<0.05. The minimal clinically important difference (MCID) was calculated with the distribution-based method of half the standard deviation of the post-operative changes for ROM, ASES, MEPI, and VAS.

## Results

Thirty-three patients with post-traumatic stiff elbow participated in this cohort. 32 of 33 patients (97%) were available at a minimum follow-up of three months (range: three months to three years). The average age was 40 years (range: 31-49 years). An elbow injury in 75% of the patients was on the dominant side (Table 2).

**Table 2:** Clinical characteristics of the patients (n=32)

Variable	Number	Percentage
Gender		
Male	21	66
Female	11	34
Side of Injury		
Right	24	75
Left	8	25
Dominant Extremity		
Right	22	69
Left	6	31

The average time from injury to arthroscopic intervention was 20 months, ranging from three months to five years. The etiologies of elbow stiffness include 17 distal humerus fractures, eight proximal ulna fractures, five proximal radius fractures, one elbow dislocation, and one heterotopic ossification after soft tissue injury around the elbow (Table 3).

**Table 3:** The etiologies of the elbow stiffness (n=32)

Variable	Frequency	Percentage
Distal Humerus Fracture	17	53
Proximal Ulnar Fracture	8	25
Proximal Radius Fracture	5	16
Elbow Dislocation	1	3
Heterotopic Ossification after Soft Tissue Injury	1	3

Range of motion

After a minimum follow-up of three months (range: three months to three years), the elbow arc of flexion extension improved from 74 ± 11 to 110 ± 15 degrees, which was statically significant. The average post-operative elbow ROM improvement in our patients was 36 degrees. The change in the arc of supination-pronation did not show any significant difference because almost all the patients did not have any limitation in supination-pronation except one patient with heterotopic ossification following nonoperative management of soft tissue injury (Table 4).

American shoulder and elbow surgeons score

The ASES score went from 69 ± 3.4 pre-operatively to 79 ± 6.3 at the final follow-up, with a mean improvement of 10 that was statistically significant (P < 0.001) (Table 4).

**Table 4:** Pre-operative and post-operative ROM, MEPI, ASES, VAS, grip strength ROM: Range of Motion; ASES: American Shoulder and Elbow Surgeons; MEPI: Mayo Elbow Performance Index; VAS: Visual Analog Scale *Minimum post-operative period was three months (range: three months to three years)

Variable	Pre-operative	Post-operative*	P-value
	Mean ± SD		
ROM	74 ± 11º	110 ± 15º	<0.001
ASES	69 ± 3.4	79 ± 6.3	<0.001
MEPI	64 ± 6	82 ± 8	<0.001
VAS	1.1 ± 0.87	0.31 ± 0.53	<0.001
Grip Strength	59 ± 3.7	60 ± 3.8	0.256

Mayo elbow performance index

The MEPI score improved from 64 ± 5.7 pre-operatively to 82 ± 8 at the final follow-up, with a mean improvement of 18 (P < 0.001) (Table 4).

Visual analog scale

At rest, the mean VAS score was 1.1 ± 0.87 (range: 0 - 3) pre-operatively and 0.31 ± 0.53 (range: 0 - 2) post-operatively. These changes were significantly different pre-operative and post-operatively (Table 4).

Grip strength

The mean grip strength was 59 ± 3.7 kg pre-operatively and 60 ± 3.8 kg post-operatively. These changes were not significantly different pre-operatively and post-operatively (P=0.256) (Table 4).

Minimal clinically important difference

The MCID values for ROM, ASES, MEPI, and VAS scores were calculated to be 7.5, 3.15, 4, and 0.265, respectively The MCID was met for all factors except VAS (Table 5).

**Table 5:** MCID values of ROM, ASES, MEPI, and VAS scores MCID: Minimal Clinically Important Difference; ROM: Range of Motion; ASES: American Shoulder and Elbow Surgeons; MEPI: Mayo Elbow Performance Index; VAS: Visual Analog Scale

Variable	Value
MCID	
ROM	7.5
ASES	3.15
MEPI	4
VAS	0.265

Complications

Post-operative complications included three transient ulnar nerve paresthesia and one superficial infection. The paresthesia was limited to the small finger and was managed conservatively by close observation while the infection was treated with oral antibiotics. We did not encounter any intra-operative complications and no surgery converted to an open release. The complication rate including minor transient events was 12%.

## Discussion

The significant finding of our study demonstrates that arthroscopic release is an effective option to restore elbow function in patients with PTES. Pre-operative ROM and patient-reported outcomes (PROs) improved significantly at the final follow-up. However, the change in grip strength was not significant. It is important to note that all patients gained some degree of motion in the direction that was limited, while we did not notice any loss of motion after arthroscopic elbow release. Preserved supination-pronation was not affected after arthroscopic release for limited flexion-extension arc.

In our experience, the complication rate was similar in the first patients compared to the last patients, however, the first surgeries were taking longer, and two patients underwent a two-stage surgery due to extravasation obscuring visualization. The second stage was performed after 2-3 days. In these patients, the posterior compartment release was performed in stage one and the anterior compartment release was done in the second stage. 

In a systematic review comparing open versus arthroscopic elbow stiffness release, Kodde et al. [21] showed that arthroscopic release is associated with fewer complications than open procedures. They reported an average complication rate of 23% (0-59%) with open arthrolysis, 5% (0-11%) with arthroscopic arthrolysis, 73% (57-88%) with open arthrolysis and external fixator, and 58% with open arthrolysis and distraction arthroplasty [21]. Complications were mostly reported as (transient) ulnar neuropathy and infection. Additionally, arthroscopy allows for better visibility of intraarticular structures and more appropriate debridement of all elbow compartments [12]. We support these findings with our improved results and low complication rate.

There is relatively small data available on PTES alone, with case numbers varying from 6-34 [9,18,20,25,26], and our sample size of 32 patients is comparable to the previous literature. Our results were consistent with the other studies showing that arthrolysis between three to six months after the index surgery results in a greater ROM improvement. It is intuitive that a longstanding stiffness leads to muscle stiffness and imbalance that despite internal arthrolysis, still the external stiffness persists. Other studies have shown the influence of biceps-triceps co-contraction in more chronic conditions than acute elbow injuries [27].

The average postoperative elbow ROM improvement in our patients was 36°, consistent with prior literature showing a 39° improvement in similar groups [14]. In a systematic review, Kodde et al. reported an average change of 51° (from an average of 52° preoperatively to 103° postoperatively) after open arthrolysis and 40° (from an average of 84° preoperatively to 124° postoperatively) after arthroscopic arthrolysis [21].

Wu et al. showed improved MEPI and reduced pain scores [9], and Bustamante-Suárez de Puga et al. [25] confirmed that arthroscopic release in PTES is an effective intervention to restore mobility in the short term. Despite the favorable results, several authors reported that the ROM does not improve in some patients [9-11] These conflicting outcomes can be explained by patient or surgeon-specific reasons. Patients with high preoperative pain levels have poorer outcomes [20]. Also, Kim and Shin [12] found that the ROM showed better improvement in patients with symptoms for less than 12 months compared to those with symptoms for a longer duration (49° vs. 30°). Arthroscopic elbow release is a technically demanding surgery involving different portals, arthroscopic retractors, ulnar nerve isolation, and avoidance of excessive intra-articular joint pressure. Thus, the surgeon’s proficiency in maneuvering within the elbow compartments as gently as possible is crucial for improving functional and (PROs) [28].

A cadaver study reported a higher complication rate when elbow arthroscopy is done by a novice surgeon [22]. The complication rate in our study was 12%, similar to those reported by other authors [9,14]. Specifically, we had three patients with post-operative transient nerve paresthesia and one superficial infection treated with antibiotics. The nerve symptoms were limited to the ulnar nerve, unlike studies that described posterior interosseous nerve [11] and median nerve [12] injuries. The reason might be our more aggressive approach to debride the ulnar nerve floor as distal as possible, causing ulnar nerve irritation; however, symptoms resolved in all three patients after an average of three to six months with no motor involvement. Our technical recommendation is always to use the shaving tool facing the bone while protecting the nerve behind it. In a large prospective study of arthroscopic elbow release, Blonna and O'Driscoll [29] showed that prophylactic decompression reduced the risk of neuropathy in nearly all cases. Furthermore, Williams et al. [30] found that ulnar nerve complaints were uncommon after elbow release in subjects with preoperative symptoms or a positive Tinel test. On the contrary, we agree with Schreiner et al. [20] that surgical ulnar nerve decompression is not routinely required as the symptoms are only transient, with patients fully recovering at three to six months follow-ups.

Surgical intervention for PTES can be offered to patients who fail to improve after three months of nonoperative treatment, which may be open or arthroscopic. Although open procedures are used widely with good functional outcomes [2,31,32] they require extensive dissection with muscle splitting, which may make recovery harder. Moreover, a review comparing complications between open versus arthroscopic tennis elbow release showed comparable outcomes with a higher infection rate after open surgery [33]. On the other hand, elbow arthroscopy provides less surgical morbidity and more ability to comfortably start physical therapy after the operation. Even though arthroscopy of the elbow carries risks due to the proximity of the portals to vital nerves and vessels, a deep understanding of the elbow's intraarticular anatomy and technique could prevent serious complications. 

Improved surgical education and instrument manufacturing have made elbow arthroscopy safe and effective; however, complications result from a lack of experience, poor technique, or a dearth of knowledge about the anatomy of the elbow [34]. Elbow arthroscopy is known to be complex [35]; in particular, precise placement of portals for optimal visualization and access to the elbow can be challenging, and the learning curve of this procedure can be steep [22]. Novice surgeons may require additional time and training to achieve proficiency in the technique, as well as mentoring from experienced surgeons [22]. Preclinical and/or “computer-based hands-on experience”, preferably fellowship-based practice, is recommended before arthroscopic elbow surgery can be safely performed on a patient [22]. As surgeons gain experience, they can increase efficiency and master elbow arthroscopy, improving patient outcomes and reducing operative time [35].

While our study provides important insights into the clinical outcomes after the arthroscopic release of PTES, it has several limitations. There is a shortage of literature regarding the arthroscopic release of elbow stiffness specifically caused by traumatic incidents. Moreover, the absence of a control group receiving alternative treatments precludes definitive conclusions on the benefits of this surgical intervention. Finally, the follow-up period may not be sufficient to objectively evaluate the clinical outcomes, even though Cefo and Eygendaal [18] reported that patients recover three months after arthroscopic release with minimal extra improvement afterward. Therefore, case-control studies with long-term follow-up should be conducted to confirm our findings and investigate more deeply the relationship between pathogenic factors and clinical outcomes of PTES.

## Conclusions

This study demonstrated that arthroscopic arthrolysis provides an effective and non-invasive treatment option for patients with PTES. Presented tips and pearls have important implications for clinical practice, as they give elbow surgeons more accurate predictions of patients' outcomes after stiff elbow arthrolysis. We believe that arthroscopic release is safe and reliable for treating patients with PTES, improving joint visibility, reducing pain, and minimizing complications.
